# AGING AND MENTAL HEALTH DURING AND AFTER THE PANDEMIC. AND OLDER ADULT SPECIALTY PSYCHOTHERAPY CLINIC FINDINGS

**DOI:** 10.1093/geroni/igad104.2128

**Published:** 2023-12-21

**Authors:** Melba Hernandez-Tejada, Deborah Little, Madeleine Desrochers, Sarly Butte, Ron Acierno

**Affiliations:** University of Texas Health Science Center at Houston, Houston, Texas, United States; University of Texas Health Science Center, Houston, Texas, United States; University of Houston, Houston, Texas, United States; University of Texas Health Science Center at Houston, Houston, Texas, United States; University of Texas Health Science Center at Houston, Houston, Texas, United States

## Abstract

A specialty clinic addressing psychological trauma secondary to elder abuse opened a few months before the pandemic began. The aim was to provide evidence-based psychotherapy for post-trauma psychological sequalae. We leveraged technology in the form of home-based telemedicine, to deliver effective treatment directly into older adults’ homes. We here present psychotherapy retention rates and clinical outcomes of this endeavor. Method: single arm, repeated measures, clinical series case study design. Outcomes: treatment initiation and completion rates; standardized measures of PTSD (PCL5), depression (PHQ9), anxiety (GAD7) and sleep (PSQI) collected at pre- and post-treatment. Results Prior to implementing this specialty clinic, the larger clinic within which it was housed saw approximately 2 older adults per year. Tailoring the program to older adults resulted in an annual census of over 100 per year. Analysis of current data indicates excellent completion rates for evidence-based psychotherapy (above 75% compared to 60% of younger patients) and clinical improvements across symptom areas in excess of one standard deviation. Discussion The success of this specialty trauma clinic for older adults experiencing violence was enhanced by a pre-pandemic decision to use telemedicine to increase reach and ease of accessing care. Initial results indicate that televideo technology was well received, was used by older adults and implemented before and during the pandemic with no issues, even for cases of severe trauma reactions such as PTSD and depression in older adults.

